# Impact of Post-Harvest Management Practices in Corn (*Zea mays* L.) Fields on Arthropods in Subsequent Soybean (*Glycine max* [L.] Merr.) Plantings

**DOI:** 10.3390/insects14010093

**Published:** 2023-01-15

**Authors:** Alan W. Leslie, Scott R. McCluen, Cerruti R. R. Hooks

**Affiliations:** 1 University of Maryland Extension, University of Maryland, Charles County, Bel Alton, MD 20611, USA; 2 Department of Entomology, University of Maryland, College Park, MD 20742, USA

**Keywords:** herbivore, natural enemies, parasitism, predation, stink bug, tillage

## Abstract

**Simple Summary:**

Soybean plantings typically consist of a diversity of herbivores and beneficial arthropods. In many instances, soybean producers rely on insecticide sprays to suppress pestiferous insects. However, these sprays may prove more damaging than beneficial. Increased vegetation diversity aims to enhance natural enemy abundance and efficacy and subsequently reduce pest populations more sustainably. The aim of this study was to investigate the impact of three post-harvest practices in corn, resulting in varying levels of plant residue remaining on the soil surface, on populations of arthropod pests and beneficials within a subsequent soybean crop. Overall, insect herbivore abundance was greater in soybean with no cover crop and/or corn plant residue. However, the abundance of predators within the soybean foliage was similar among plots with plant residue or most residue removed via plowing. Among ground predators, spider activity density was greatest in soybean where residue was plowed under. Stink bug egg mortality due to predation and parasitism were inconsistent among treatments. Findings suggest that post-harvest practices investigated during this study will have a similar influence on most ground and foliar arthropods. However, post-harvest practices that limit tillage and maintain plant residue on the surface promote long-term sustainable ecosystems that benefit society.

**Abstract:**

There is increased adoption of cover cropping and conservation tillage in the USA. Many farmers view these practices as methods for improving their soils. However, different cover cropping and tillage practices conducted post-harvest can have a disparate impact on arthropods within the subsequent cash crop. Field experiments were conducted during 2017 and 2018 at two experimental sites to examine the influences of different post-harvest practices following corn (*Zea mays* L.) harvest on pests and beneficials in subsequent soybean [*Glycine max* (L.) Merr.] plantings. Experimental treatments included: (1) tillage via chisel plow (CP), (2) no-tillage in which corn residue/stubble remained on the soil surface (CS), and (3) planting a cover crop into corn residue (CC) following corn harvest. Overall, insect herbivore abundance was greater in the CP treatment. Foliar predator numbers were similar among treatments or of greater abundance in CP. The activity density of epigeal insect predators varied according to site and feeding guild. However, spider activity density was greatest in CP. Stink bug egg mortality due to predation and parasitism varied among treatments. However, the percentage of stink bug eggs that hatched was greatest in the CC during both years. Findings suggest that post-harvest practices investigated during this study will have a similar influence on most epigeal and foliar arthropods in soybean.

## 1. Introduction 

Global soybean [*Glycine max* (L.) Merr.] production in 2017/2018 was estimated at ~ 337 million metric tons [1]. Notwithstanding, it has been suggested that farmers worldwide must increase crop production over the coming decades to keep pace with a rapidly growing international population [2]. Consequently, much of soybean research has centered on methods to increase yield [3]. Biophysical limits on crop growth are an important factor in determining yield potentials [2]: however, improving crop stress tolerance [4] and protecting crops from disease, insect and weed pests must be done concomitantly to improve crop yields [5]. Plant diversification in the form of greater vegetation complexity and/or diversity has been proposed as a natural and eco-friendly method to regulate agricultural pests and create resilient farming systems [6,7]. As such, a captious complaint of monoculture cropping systems is that reduced habitat complexity causes these systems to be more vulnerable to pest outbreaks [8]. In monoculture cropping systems, beneficial organisms are thought to be adversely affected by a lack of refuge, alternative prey and food sources, and other conditions required for optimal performance [8]. In variance, pestiferous organisms are forecasted to benefit from simple crop plantings [9]. The complexity of agroecosystems can be heightened via the added inclusion of plant residue and living plants [9,10,11,12]. Cover crops can influence arthropod communities within cropping systems by reducing herbivore colonization and enhancing natural enemy abundance, and can do so as a living mulch or organic residue [10,11,12].

Cover cropping with plants such as rye (*Secale cereal* L.), Vetch (*Vicia villosa* L.), and wheat (*Triticum aestivum* L.) has been historically researched for its potential to provide arable lands with soil quality services such as reducing erosion [13], as well as increasing soil organic matter [14,15] and nutrient levels [16]. Retention of cover crop residue on the soil surface as part of conservation tillage has also been researched for its potential to prevent weed establishment [17,18,19]. Recently, cover crops have become of greater interest for use in manipulating organisms (e.g., nematodes, arthropods, and weeds) that may impose stress on field crops and subsequently reduce yield [20,21,22,23]. Studies have shown that cover crops can have a positive, negative or neutral effect on organisms in field crop agroecosystems [24,25,26,27,28,29]. Notwithstanding, a better understanding, of interactions between cover crops and living organisms is necessary to maximize their value to field crop systems [30].

Among some common post-harvest practices is conventional tillage, leaving the plant residue undisturbed (no-tillage), or planting a winter cover crop [13]. Conventional tillage with a chisel plow facilitates breaking compact layers and mixing large amounts of soil, and as a result, heavy plant residue can be effectively buried. However, tillage systems can negatively affect soil and water quality, as well as have a significant effect on nitrogen dynamics and nitrogen availability for plants [31]. No-tillage, which involves planting crops without tillage, limits soil disturbances. This approach has been primarily researched from the perspective of conservation agriculture [32]. Conservation tillage practices, which are often used with cover cropping, improve soil quality and decrease costs, labor, and soil erosion [33,34,35]. These benefits have impelled many soybean farmers to adopt conservation tillage practices in recent years [36]. 

Different post-harvest tillage and cover cropping practices could result in varying amounts of residue remaining on the soil surface within fields [13]. This variation in complexity could influence arthropod communities (herbivores, natural enemies) within a subsequent crop discordantly [10,27,29]. Nevertheless, how post-harvest field practices following a corn planting impact herbivorous pests, natural enemies, and yield within the subsequent soybean crop have not been well investigated. To address this paucity, we examined and compared the impact of three commonly used post-harvest practices in corn fields (tillage, no action, and planting a cover crop following corn harvest) on arthropod pests and beneficials, and yield within a subsequent soybean crop. It was hypothesized that herbivorous insect populations would be less abundant and arthropod natural enemies would be of greater abundance and have greater efficacy in soybean habitats consisting of more plant residue on the soil surface.

## 2. Materials and Methods 

### 2.1. Experimental Treatment and Plot Layout

Field experiments were conducted at the Western Maryland Research and Education Center in Keedysville (WM, 39°30′34.271″ N, 77°44′0.128″ W, 150 m a.s.l.) and the Central Maryland Research and Education Center in Beltsville (CM, 39°0′44.7114″ N, 76°49′32.5626″ W, 51 m a.s.l.) Maryland during 2017 and 2018. Treatments were replicated four times and arranged in a randomized complete block design. Each block consisted of three post-harvest treatment methods following corn harvest: (1) conventional till in which plots were chisel plowed to remove corn residue (CP), (2) undisturbed in which corn residue/corn stubble was allowed to remain following harvesting (CS), and (3) cover crop was planted into the corn residue following harvest (CC). The CC treatment consisted of a rye/crimson clover (*Secale cereale* L. + *Trifolium incarnatum* L.) mixture planted at 9 and 76 kg/ha, respectively. Each plot was (10.7 × 9.1 m) and was separated by 9.1 m of regularly mowed, natural vegetation. Individual plots consisted of 12 soybean rows, which were no-till planted at a rate and inter-row spacing of 62,951 seeds ha^−1^ and 76.2 cm, respectively.

### 2.2. Management Tasks

Following corn harvest, conventionally tilled plots (CPs) were chisel plowed, rye/crimson clover mixture was drilled into corn residue in CC plots, and no post-harvest operations were performed in the CS plots. To prepare plots for soybean planting at the Keedysville site, CP plots were chisel plowed, disked, and the soil smooth with one pass of a roller harrow. At the Beltsville site, CP plots were disked twice, and a single pass was performed with the roller harrow. Burndown of cover crops and weeds was achieved in CM using a combination of 2,4-D ester at 0.40 kg ai ha^−1^ and paraquat at 1.05 kg ai ha^−1^ in both years. At WM, cover crops and weeds were terminated using 2,4-D ester at 0.53 kg ai ha^−1^ and paraquat at 1.26 ka ai ha^−1^ in 2017 and 2,4-D ester at 1.06 kg ai ha^−1^ and glyphosate at 1.89 kg ae ha^−1^ in 2018. Residual herbicides applied preemergence included S-metolachlor at 1.42 kg ai ha^−1^ and sulfentrazone (0.20 kg ai ha^−1^) + cloransulam methyl (0.025 kg ai ha^−1^) at both sites and years. The soybean variety (Pioneer P37T09L Maturity 3.7) planted was genetically modified to be tolerant to glufosinate. As such, postemergence weed control included glufosinate at 0.66 kg ai ha^−1^, with the addition of clethodim for better grass weed control. At CM, the postemergence spray included glufosinate and clethodim at 0.07 kg ai ha^−1^ in 2017 and clethodim only at 0.07 kg ai ha^−1^ in 2018. At WM, the postemergence spray included glufosinate only in 2017 and glufosinate plus clethodim at 0.28 kg ai ha^−1^ in 2018. To estimate yield at each site, all rows in each plot were harvested with a small plot combined at 13 to 14% moisture. The specific timing of field operations is indicated in Table 1.

### 2.3. Foliar Sampling of Pests and Beneficial Arthropods

Arthropods within the soybean foliage were sampled weekly with the use of a 38.1 cm diameter canvas sweep net for relative population estimates. A collected sweep sample consisted of two sets of five sweeps performed down and across two randomly selected row areas at a sweeping width of 1.0 m. Rows were randomly chosen for each sampling occasion. Sampling at both sites began approximately when soybean was in the beginning bloom (R1) stage of development, which occurred approximately four weeks after planting. Sampling continued each year until the early senescence or full seed (R6) stage and was conducted weekly between the hours of 8:00 a.m. and 12:00 p.m. In 2017, eight sweep samples were collected weekly at each site from 30 June to 5 September; and in 2018, six samples were collected weekly from 11 July to 29 August. Arthropods were transferred into plastic Ziploc^®^ storage bags (S.C. Johnson & Son, Rascine, WI, USA) and temporarily placed in a portable cooler while in the field. They were then transported to the laboratory and temporarily stored in a freezer at ~−20 °C for later identification and counting. Arthropod samples were initially sorted on white trays under a 10× desktop magnification lamp. Soybean leaves were brushed with a small horsehair brush to remove micro-parasitoids and other arthropods that were found in the plastic bags. Specimens were later identified to the family level and placed in 85% ethyl alcohol for storage.

### 2.4. Pitfall Trap Sampling

Epigeal predators were sampled at both sites weekly during the 2018 growing season by placing one pitfall trap in the inter-row area between two center rows. Each trap consisted of two 355 mL clear plastic cups. The top cup was placed inside the bottom cup, and approximately 60 mL of ethylene glycol was poured inside. The bottom cup was buried so that the top of the upper cup was just below the soil surface, and holes were drilled into the bottom cup to allow rainwater to drain. A 30 cm × 30 cm plastic cover supported by three 8 cm carriage bolts was centered ~2 cm above each cup and fastened by pushing the bolts into the soil to prevent weather and wildlife interference. Traps were replaced weekly over 7-days intervals from May through September. Captured arthropods were vacuum filtered and rinsed over fine organdy cloth in the laboratory to remove any ethylene glycol. Samples were then stored in 70% alcohol, pending further processing. Trap contents were transferred to Petri dishes and viewed under a dissecting microscope (Leica M60 stereo microscope, Leica Microsystems Inc., Buffalo Grove, IL, USA), where specimens were identified to the lowest possible taxonomic level.

### 2.5. Natural Enemy Efficacy

To quantify treatment impact on natural enemy efficacy, the kudzu bug (KB), (*Megacopta cribraria*; Hemiptera: Plataspidae) predatory spined soldier bug (SSB), (*Podisus maculiventris; Hemiptera: Pentatomidae*) and several herbivorous stink bugs were monitored: the brown marmorated stink bug (BMSB), (*Halyomorpha halys*; *Hemiptera: Pentatomidae*) the brown stink bug (BSB); (*Euschistus servus*; *Hemiptera: Pentatomidae*) the green stink bug (GSB), (*Chinavia hilaris; Hemiptera: Pentatomidae*) the red-shouldered stink bug (RSSB), (*Thyanta custator*; *Hemiptera: Pentatomidae*) and harlequin bug (HB) (*Murgantia histrionica*; *Hemiptera: Pentatomidae*).

Stink bug egg mortality due to predation and parasitism was quantified at the CM study site during the 2017 and 2018 growing seasons. Their numbers were not high enough to monitor at the WM site. To quantify egg mortality, soybean plants in each plot were searched several days weekly once eggs appeared. Plants were sampled for approximately 14 weeks. If a stink bug egg mass was found, flagging tape was tied to the stem just below the trifoliate leaf containing the egg mass, and a circle was drawn around the egg mass with a permanent marker. Eggs were identified to species, counted, recorded, and checked several days weekly to determine their fate. Eggs were classified as (1) hatched, in which stink bug nymphs emerge; (2) missing, in which eggs disappear from the surface of the leaf; (3) mortality unknown, in which eggs did not hatch and showed no signs of predation or parasitism; (4) mortality due to parasitism, in which eggs were parasitized and (5) mortality due to predation, in which eggs were shrunken or collapsed and/or chewed. Eggs attacked by chewing predators were distinguished from those attacked by sucking predators. During each sampling occasion, if predators or parasitoids were found on or in the vicinity (next to the egg mass), their identity and activity were recorded. Eggs that did not hatch were taken to the laboratory for further screening under a dissecting microscope (Leica M60 stereo microscope, Leica Microsystems Inc., Buffalo Grove, IL, USA).

### 2.6. Statistical Analysis

This study produced five datasets for analysis: (a) sweep net sampling, (b) pitfall trap, (c) stink bug and kudzu bug egg fate, and (d) crop yield data. The total abundance of all arthropods and their abundances according to feeding guild, as well as soybean yield data, were recorded for each treatment and analyzed to determine the impact of treatment (three post-harvest practices in corn fields) on pests, beneficials, and crop yield in soybean. Data collected for each year (2017 and 2018) and study site [Beltsville (CM) and Keedysville (WM)] were analyzed separately.

For sweep samples, the abundance of all arthropods (all individuals collected in a sample, regardless of feeding guild) was calculated for each block and treatment, averaged across dates. The Mean abundance of arthropods was also calculated according to the feeding guild for each block and treatment, averaged across dates. Generalized linear mixed models (GLMMs) using Poisson distribution (for count data) were fitted by maximum likelihood (Laplace Approximation) to determine differences in arthropod abundances among treatments. Treatment was treated as a fixed effect, and date and block as random effects. The pitfall trap data were analyzed similarly.

For stink bug egg fate data, the percentage of stink bug egg mortality due to predation and parasitism and percentage that hatched were analyzed separately for GSB, BMSB, and BSB. Other stink bug species sampled did not occur in high enough numbers to warrant a separate analysis. All stink bug species were also analyzed as a group (Pentatomidae). For the kudzu bug, egg fate data was characterized and analyzed as mortality due to predation and unknown factors or successfully hatched. GLMMs using Poisson distribution were fitted by maximum likelihood to determine treatment differences in egg mortality due to natural enemy activity and successful hatching. Treatment was treated as a fixed effect, and date, block, and insect species were included in the model as random effects. Parasitism and predation rates, as well as the proportion of hatched individuals, were also compared across different stink bug species.

Total crop yield was calculated as a sum of yield values per each block and treatment. Three linear mixed models (LMMs) were fitted by REML to determine the differences in the total crop yield among treatments. Treatment was treated a as fixed effect, and blocks were included in the model as random effects. GLMMs and LMMs were performed using lme4 package in R [37]. For each significant term from GLMMs, multiple means comparisons were performed by computing estimated marginal means (aka least-squares means) using emmeans package in R.

## 3. Results

### 3.1. Foliar Sampling of Pests and Beneficial Arthropods

A total of 3889 arthropods representing 100 different taxa (families) were collected from both locations during the two-year study. A total of 1932 arthropods were collected from CM (1107 in 2017 and 825 in 2018), and 1957 arthropods were collected from WM (1282 in 2017 and 675 in 2018). A total of 1795 insect herbivores were assigned to feeding guilds, which represented 46% of all arthropods collected (14% and 32% for chewing and sucking herbivores, respectively) and represented 31 families. The most abundant chewing herbivores were Chrysomelidae (31%), Scarabaeidae (22%), and Acrididae (14%). Among sucking herbivores, the most abundant families were Cicadellidae (40%), Miridae (33%), and Thysanoptera (9%). A total of 824 insect predators were collected, which represented 21% of all arthropods collected (4% and 17% for chewing and sucking predators, respectively) in 22 families. The most abundant chewing predators were Formicidae (45%) and Coccinellidae (37%). Among sucking predators, the most abundant families were Anthocoridae (43%), Nabidae (29%), and Geocoridae (18%).

In 2017, at CM, total arthropod abundance was greater in CP than CS and CC treatments (p_adj_ = 0.002 and p_adj_ = 0.001 respectively), whereas no differences were detected between CC and CS (Table 2). A greater number of arthropods was observed for most feeding guilds in CP treatment (Table 2). The total abundance of all herbivores (chewing and sucking) was greater in CP than in CS and CC treatments (CC–CP: p_adj_ = 0.01, CP–CS: p_adj_ = 0.004). The number of chewing herbivores was also higher in CP than in CS and more sucking herbivores were found in CP than in CC treatment. The total abundance of all predators was higher in CP than CC treatment (p_adj_ = 0.01); however, no differences were detected for chewing and sucking predators when analyzed separately.

In 2017, at WM, no differences in the total abundance of arthropods were detected among treatments (Table 2). The abundance of different feeding guilds, however, varied among treatments. Similar to the CM location, the total abundance of all herbivores was higher in CP than in CC and CS treatments (p_adj_ = 0.02 and p_adj_ = 0.03, respectively; Table 2). and numbers were similar among CC and CS treatments. Similar results were observed for sucking herbivores, whereas no differences among treatments were detected for chewing herbivores. The total abundance of all predators, as well as chewing and sucking predator guilds, did not differ among treatments (Table 2).

In 2018, at the CM location, no differences were detected among treatments in the total abundance of arthropods. However, the total number of all herbivores and chewing herbivores were greater in CP than in CS (p_adj_ = 0.003 and p_adj_ = 0.04, respectively; Table 2). Similar numbers were observed in each treatment for the total number of predators as well as chewing and sucking guilds (Table 2). In 2018, in WM, total arthropod abundance was greater in CP than in CC treatment (p_adj_ = 0.009). No differences were found in the total abundance of herbivores or their abundance by feeding guild (Table 2). The total abundance of all predators was higher in CP than CC (p_adj_ = 0.003); and in CS than CC (p_adj_ = 0.0001; Table 2) treatment. The abundance of chewing predators was higher in CS than CP and in CS than CC treatment (p_adj_ = 0.0005 and p_adj_ = 0.02, respectively; Table 2). No differences were detected among treatments in the number of sucking predators.

A total of 487 parasitoids and 380 spiders were collected, which comprised 13% and 10% of all arthropods collected, respectively. The most abundant parasitoids encountered were Platygastridae (38%). Spiders were primarily represented by the following families: Oxyopidae (23%), Salticidae (20%), Lycosidae (16%), Thomisidae (16%), and Linyphiidae (14%). In 2017 and 2018, in CM and WM locations, the mean abundance of parasitoids and spiders did not differ among treatments (Table 2).

### 3.2. Pitfall Trap Sampling

A total of 1311 arthropods representing 86 different taxa (families) were collected from both locations. This included 551 arthropods from CM and 760 arthropods from the WM location. Of these, a total of 180 insect herbivores comprised of 23 families were collected. Insect herbivores represented approximately 14% of all arthropods collected (9% and 4% for chewing and sucking herbivores, respectively). The most abundant chewing herbivores were Nitidulidae (34%), Monotomidae (20%), and Scarabaeidae (20%). Among sucking herbivores, the most abundant family was Miridae (48%). A total of 174 insect predators comprised of 10 families were collected. Insect predators represented 13% of all arthropods collected (11% and 2% for chewing and sucking predators, respectively). The most abundant chewing predators were Staphylinidae (48%) and Carabidae (40%). Sucking predators were represented mostly by two families, Lampyridae (50%) and Cantharidae (47%). A total of 48 parasitoids and 183 spiders which comprised 4% and 14% of all arthropods collected, respectively, were sampled. The most abundant parasitoids encountered were Scelionidae (48%), and the most abundant spiders sampled were Lycosidae (75%).

In 2018, the total abundance of invertebrates differed among treatments at the CM and WM locations (Table 3). At the CM location, the highest number of invertebrates was observed in CP treatment (p_adj_ < 0.001). The abundance of all predators and chewing predators (including species from the family Carabidae, which was also analyzed separately) was higher in CC and CP than in CS treatment (p_adj_ < 0.001 and p_adj_ = 0.002, respectively; Table 3), while no differences were observed between CC and CP treatments. The abundance of sucking predators varied across treatments and was higher in CC and CP than in CS treatment (p_adj_ < 0.001). The abundance of cumulative (sucking + chewing) herbivores and chewing herbivores were higher in CC than in CP and CS treatments (p_adj_ < 0.001 and p_adj_ = 0.005, respectively); the highest abundance of sucking herbivores, however, was detected in CS treatment (p_adj_ = 0.002). No differences in the abundance of parasitoids were recorded among treatments. The abundance of spiders was higher in CP than in CC and CS treatments (p_adj_ = 0.004); and no differences were detected between CC and CS treatments (Table 3).

At the WM location, the highest number of invertebrates was observed in the CC treatment (p_adj_ < 0.001; Table 3). The abundance of cumulative and chewing predators was higher in CC than CP and CS treatment (p_adj_ < 0.001 and p_adj_ = 0.017, respectively); and no differences were observed between CS and CP treatments. The abundance of sucking predators, however, was higher in CC and CP than CS treatment (p_adj_ < 0.001). The highest abundance of cumulative herbivores and chewing herbivores was recorded in CP treatment (p_adj_ < 0.001 and p_adj_ = 0.015, respectively; Table 3), and no differences for sucking herbivores were observed. The abundance of parasitoids and spiders were similar among treatments.

### 3.3. Natural Enemy Efficacy

At the CM location, a total of 9365 stink bug eggs were monitored as part of the natural enemy efficacy survey. This included 4031 eggs in 2017 and 5334 in 2018. Of these, egg mortality due to parasitism, in both years, was recorded for 2544 eggs (27% of all stink bug eggs); egg mortality due to predation was recorded for 1193 eggs (13%); and a total of 4908 eggs (52%) successfully hatched. For both study years, a total of 3097 stink bug eggs (33% of the total number of eggs found) were encountered in CC, 3285 (35%) in CP and, 2983 eggs (32%) in CS treatment.

In 2017, the percentage of stink bug egg mortality due to parasitism was lower in CS than in CP and CC treatments (p_adj_ = 0.0005 and p_adj_ = 0.003, respectively; Table 4). However, no differences in percent egg mortality due to predation were observed among treatments. The proportion of hatched eggs, however, was significantly higher in CC than in CP and CS treatments (p_adj_ = 0.005 and p_adj_ = 0.002, respectively; Table 4). In 2018, percent egg mortality due to parasitism differed significantly among treatments. The percentage of parasitized eggs was lower in CC than in CP and CS treatments (p_adj_ < 0.0001). The percentage of egg mortality due to predation and proportion that hatched were higher in CC than in CP and CS treatments (p_adj_ = 0.0001) and were greater in CC and CS than CP (p_adj_ = 0.01 and p_adj_ = 0.0006, respectively; Table 4).

The most abundant stink bug eggs encountered were GSB (7229), BSB (1446), and BMSB (513), which comprised 77%, 15%, and 5% of all stink bug eggs, respectively. Because the greatest number of eggs found represented GSB and BSB, these species were analyzed separately according to treatments. During both studies years, differences in parasitism rate, predation rate, and proportion of hatched eggs for GSB and BSB were similar among CC, CP, and CS treatments.

In 2017, the analysis of egg mortality among stink bug species across all treatments indicated that the parasitism rate (calculated as a proportion of parasitized eggs) for *H. halys* (BMSB) was lower than for *E. servus* (BSB) and *P. maculiventris* (SSB) (p_adj_ < 0.0001; Table 5). In addition, the parasitism rate for *C. hilaris* (GSB) was lower than BSB and SSB (p_adj_ < 0.0001). The predation rate was higher for BMSB than BSB, GSB, and SSB (p_adj_ = 0.0001, p_adj_ = 0.0005, and p_adj_ = 0.004, respectively). The proportion of hatched eggs was higher in GSB than in BSB and SSB (p_adj_ < 0.0001 and p_adj_ = 0.01, respectively). Egg parasitism and predation rate, and the proportion of hatched eggs were similar among other stink bug species. In 2018, the parasitism rate was lower for GSB than BSB (p_adj_ < 0.0001; Table 5). The proportion of hatched eggs was higher in GSB than in BSB (p_adj_ < 0.0001). Rates of parasitism and predation, and the proportion of hatched eggs were similar among other stink bug species. *M. histrionica* (HB) and *T. custator* (RSSB) were not included in the analysis due to their small sample size.

A total of 3460 KB eggs were found (1640 in 2017 and 1820 in 2018). In 2017 and 2018 the percentage of kudzu bug (KB) egg mortality and the proportion of hatched eggs were similar among treatments (Table 6). During the two years, egg mortality among treatments ranged from approximately 5.8% to 19.2% and the proportion of hatched eggs ranged from 75.1% to 93.2%.

### 3.4. Crop Yield

In 2017, soybean yield was similar among treatments at the CM location. In WM, the yield was lower in the CC than in CP and CS treatments (p_adj_ = 0.017 and p_adj_ = 0.018, respectively; (Table 7), whereas no differences were observed between CP and CS treatments. In 2018, crop yields were similar among treatments at both study sites.

## 4. Discussion

### 4.1. Summary of Arthropod and Crop Yield Responses

During this two-year field study, we evaluated the influences of three post-harvest field practices following corn on arthropod populations and crop yield in a subsequent soybean planting. It was hypothesized that the added vegetation diversity afforded by the corn residue/stubble (CS) or corn and cover crop residue (CC) would result in a greater abundance of beneficials and a lower number of insect herbivores compared to plots where the residue was removed via conventional tillage [chisel plow (CP)] after the corn was harvested. In addition, we hypothesized that the abundance of beneficial arthropods would be greater in habitats containing corn and cover crop residue than plots containing just corn residue. However, the findings mostly did not support our suppositions. Corn residue is an important overwintering site for numerous predaceous insects [38]. As such, the addition of cover crop to the corn residue may not have altered the habitat enough to have a marked influence on epigeal predators’ activity density. Pitfall trap catches are also influenced by arthropod movement, among other factors, and are not an adequate measurement of population size [39]. Thus, it is feasible that the added residue may have interfered with ground arthropod movement resulting in lower capture rates in CC and CS habitats. In some instances, the responses of herbivores and beneficial arthropods agreed with our supposition, and on other occasions, their response was at variance with what we hypothesized. Specifically, the abundance of some foliar pests, such as chewing and sucking herbivores, was greater in the CP treatment. However, the abundance of chewing and sucking predators was, in some instances, higher in CP treatment. Pitfall trap captures of spiders were consistently lower in CC than in CP treatment. The percentage of Pentatomidae (stink bug) eggs that successfully hatched was greatest in CC treatment. Soybean yield was only influenced by treatment at the WM site in 2017. During which, the yield was lower in the CC than in CS and CP treatment.

### 4.2. Epigeal Predators

Similar to some previous findings, results from this study showed no effect or a variable response of cover crops and residue disturbances on arthropod abundance in soybeans [23,40,41]. Overall, an increased abundance of all arthropods (cumulative feeding guilds) was recorded in the CC treatment. However, a positive effect of CC treatment on the activity density of beneficial arthropods was only observed for total and chewing predators collected from pitfall traps at the WM location. It is possible that the decreased abundance of foliar and ground chewing predators encountered in 2018 at the WM location in the CP treatment demonstrates the indirect effects of chisel plowing operation on beneficial arthropods through habitat deterioration and changes in food resources [42]. A similar positive effect of cover crops on chewing ground predators was described in a separate study [43]. Using five treatment systems, bare fallow, soybean, and three cover crop combinations (mustard/buckwheat/canola; oat–pea/rye–hairy vetch; and oat/red clover), authors of that study detected an increased activity density of two carabid species, *Amara aenea*; Coleoptera: Carabidae) and *Harpalus pensylvanicus*; (Coleoptera: Carabidae) in pitfall traps located in cover crop compared to bare fallow and soybean systems [43].

A greater abundance of epigeal predators is often associated with a lack of soil disturbances and increased complexity provided by plant residue [44]. However, in our study, there was a decreased abundance of chewing and sucking predators encountered in pitfall traps located in the CS treatment, which was left undisturbed after corn harvesting. An earlier study also found no adverse effects of chisel plowing on the activity density of carabid beetles compared to undisturbed control plots [42]. Notwithstanding, our findings contrast an investigation that found a higher number of predators in pitfall traps located in no-tillage compared to conventional tillage systems [33]. These conflicting findings suggest that the influence of conventional tillage operations on carabid beetles is not unequivocal, as some authors reported higher activity density [44] and other authors reported lower [45] or no discernable differences [46] compared to no-till systems. These variable findings may be partially contributable to arthropod species differences among study sites. Tillage impacts on arthropods may vary according to species as some may have greater sensitivity to tillage and, as such, have a more discernible response to various tillage operations. For example, a study that investigated the impact of the moldboard plow, chisel plow, and rotary tillage operations on four carabid weed seed predators found just one species was impacted by all tillage types, demonstrating species specific sensitivity to tillage [42]. During our study, arthropods were mainly grouped according to the feeding guild. As such, treatment impact on specific-species would have gone undetected. Similar carabid activity density response to contrasting residue management protocols encountered during our investigation may suggest that plant residue, whether partially incorporated into the soil or left on the surface, can change the soil biota such that both practices encourage similar numbers of ground predators [47].

Spiders appeared to be undisturbed by tillage and uninfluenced by residue management during the current study. The activity density of spiders was greatest in the CP treatment at CM and greater than CC at the WM site and similar in CC and CS habitats. However, a review investigating the impact of agricultural diversification on spiders found that spider abundance was increased in 63% of studies reviewed and that they can be increased by mulching and reduced tillage [48]. Further, a study investigating arthropod overwintering strategies in corn fields found that 24 spider species used corn residue as an overwintering refuge [38]. Thus, it is more defensible that spider activity density would be similar in CC and CS habitats than being greater in CP habitats. Notwithstanding, in variance to our findings, a study investigating the impact of tillage on spiders in sugar beets, *Beta vulgaris* (L.) following corn cultivation showed that spider activity density was higher in the reduced-tillage (zone-tillage) than moldboard tilled plots [49]. It is important to note that prior to the zone-tillage and moldboard operations, corn stalks in all plots were chopped by lightly disking the study site, indicating that zone-tilled plots received some minimum disturbances. Another study investigating the impact of tillage operations and maize residue on spiders revealed that a conventional tillage system consisting of disk plowing followed by disc harrowing had an adverse effect on ground-dwelling spiders, while no-tillage and the retention of plant residue had a positive effect on ground and plant foraging spiders [50]. Other studies have also disclosed fewer spiders in conventional than no-tillage systems [51,52]. However, a study investigating two cover crop termination practices (roller crimper vs. green manuring with a disk harrow) on ground predators showed that green manuring increased spider abundance [53]. The authors proposed that spiders responded to the enhanced detrital food chain that occurred after cover crop residue was incorporated into the soil. Spiders may have responded similarly during the current study, as the incorporation of residue is comparable.

### 4.3. Parasitoid Abundance and Efficacy

During the current study, parasitoid abundances were similar in CP and other treatments. Further, there was no monotonous effect of post-harvest practices on stink bug egg mortality due to parasitism or predation, suggesting that the tillage and residue practices conducted during the current study will not influence overall stink bug egg mortality due to natural enemy activity. Stink bug egg mortality caused by natural enemies, and unknown mortality factors can be high [54,55,56,57], suggesting that habitats with greater structural complexity may not be required to enhance stink bug natural enemy efficiency. Still, it is tenable that tillage operations can influence parasitoid and predator numbers and their impact on host and prey, especially species that use the soil as a refuge. For example, inversion tillage conducted in oilseed rape, *Brassica napus* crop, adversely impacted the survival and emergence rates of parasitoids overwintering in the soil [58]. In addition, a field investigation found that crimson clover or rye cover crops reduced infestations of (*Helicoverpa zea*; Lepidoptera: Noctuidae) and (*Heliothis virescens*; Lepidoptera: Noctuidae) via augmented predation by the red imported fire ant (*Solenopsis invicta*: Hymenoptera: Formicidae) and minute pirate bug, (*Orius insidiosus*; Hemiptera: Anthocoridae) in cotton [59]. The authors proposed that intercropping cotton into live strips of crimson clover was responsible for relaying *O. insidiosus* onto cotton plants. During the current study, we found an increased percentage of kudzu bug eggs hatched in soybean plots with corn residue (CS) than in plots with corn and cover crop residue (CC). The significance of this is uncertain as kudzu bug populations were low or absent from the study sites. However, in another study increased kudzu bug infestations were observed in conventional tillage plots [60].

### 4.4. Crop Yield

A global meta-analysis conducted to determine the impacts of no-tillage relative to conventional tillage operations on yield found that crop type influenced crop yield the greatest in no-till systems [32]. Studies evaluating the impact of rotation and no-tillage operations on soybean yield indicated that there is a yield benefit when soybean is rotated with corn [61,62], and that grain yield does not decrease in no-till systems even if early season growth of soybean is reduced [63]. Still, an extensive literature review comparing corn and soybean yields in no-till and conventional fall tillage systems in the US and Canada showed that differences in soybean yield between the two systems were negligible [64]. We proposed in the current study that yield would be greater in no-tilled plots as natural enemies’ efficiency will be enhanced in plots with greater residue. However, only in WM during study year 1, soybean yield differed among treatments. During that time, the yield was lowest in the CC treatment. Although not explicitly measured in this study, the yield reduction in the CC treatment was likely due to poor stand establishment caused by poor seed placement at planting in the high-residue plots.

## 5. Conclusions

Conservation practices of leaving all corn residue on the soil surface or planting a rye/crimson clover cover crop mixture in corn residue did not have a constant influence on the abundance and activity density of beneficials in subsequent soybean plantings compared to using a chisel plow. The two conservation practices also did not enhance the biological control services of stink bugs, as evidenced by mostly similar or greater mortality due to predators and parasitoids in chisel plowed than the no-tilled soybean habitats. Notwithstanding, during this study, chisel plowing was deployed to prepare the soil as this is a more widely used post-harvest operation on northeastern US farms. However, compared to full tillage inversion, chisel plowing preserves more vegetation on the soil surface, which can serve as a refuge for epigeal arthropods and protect them from desiccation. As such, more intensive tillage operations such as moldboard plowing and using tillage equipment that provides near or full burial of residue post-harvest can be more damaging to the soil biota and as such, may yield greater differences.

Studies considering the effect of no-tillage and plant residue on arthropods should examine different crop residues, and cover crop species, as effects of plant residue on arthropods may differ according to species. Further, the influences of post-harvest tillage operations and cover cropping practices on arthropods should be evaluated over time as their effect on arthropod communities may not be immediate (within a single growing season) and, as such, may change over several field seasons. Although the no-till residue management practices used in this study did not result in greater soybean yield, it is important to note that land management practices that limit tillage and maintain crop residue on the surface promote long-term sustainable ecosystems that benefit society [65].

## Figures and Tables

**Table 1:** Timing of field operations for the 2017 and 2018 field experiments.

Year	Location	Task	Date
2016	Beltsville	Post-harvest treatments applied	4 October
2016	Keedysville	CP plots chisel plowed	23 September
		Cover crop planted in CC plots	26 September
2017	Beltsville	CP treatment ground prepared	2 May
		Cover crop/weed burndown	10 May
		Soybean planted	22 May
		Postemergence herbicide applied	8 July
		Soybean harvested	21 October
2017	Keedysville	CP plots prepared for planting crop	2 May
		Cover crop/weed burndown	10 May
		Soybean planted	22 May
		Postemergence herbicide applied	8 July
		Soybean harvested	25 October
2017	Beltsville	Post-harvest treatments applied	27 October
2017	Keedysville	Post-harvest treatments applied	27 October
2018	Beltsville	CP treatment ground prepared	26 May
		Cover crop/weeds terminated	25 May
		Soybean planted	29 May
		Postemergence herbicide applied	19 July
		Soybean harvested	1 November
2018	Keedysville	CP treatment ground prepared	29 May
		Cover crop/weed burndown	29 May
		Soybean planted	29 May
		Postemergence herbicide applied	19 July
		Soybean harvested	26 October

Treatments: CC denotes cover crop planted in corn residue/stubble and CP represents chisel plowed (conventional tillage) plots.

**Table 2:** Mean abundances of foliar pest and beneficial arthropods and their main feeding guilds sampled by sweep net in soybean during 2017 and 2018 at two field sites, Beltsville [Central Maryland (CM)] and Keedysville [Western Maryland (WM)], across three post-harvest treatments following corn harvest. For the three treatments, CC denotes cover crop planted in corn residue/stubble; CP represents chisel plowed (conventional tillage) plots; and CS (corn stubble) no action. Mean values and standard errors (Mean ± SE) and results of statistical comparisons via fitted GLMMs are reported.

Location/ Year/ Feeding Guild	Treatment	CM		WM	
		2017	2018	2017	2018
		Mean ± SE	Mean ± SE	Mean ± SE	Mean ± SE
all arthropods	CC	22.66 ± 2.21 ^a^	28.71 ± 2.46 ^a^	26.92 ± 1.70 ^a^	16.63 ± 1.64 ^a^
	CP	26.84 ± 2.08 ^b^	29.25 ± 3.05 ^a^	29.59 ± 2.22 ^a^	20.25 ± 1.48 ^b^
	CS	22.59 ± 2.09 ^a^	26.46 ± 3.55 ^a^	27.66 ± 2.22 ^a^	19.42 ± 1.71 ^ab^
all predators	CC	2.47 ± 0.41 ^a^	1.80 ± 0.16 ^a^	1.79 ± 0.18 ^a^	1.87 ± 0.19 ^a^
	CP	2.92 ± 0.37 ^b^	1.68 ± 0.12 ^a^	1.70 ± 0.13 ^a^	2.32 ± 0.26 ^b^
	CS	2.70 ± 0.45 ^ab^	1.73 ± 0.17 ^a^	1.91 ± 0.19 ^a^	2.58 ± 0.45 ^b^
chewing predators	CC	1.71 ± 0.57 ^a^	1.71 ± 0.57 ^a^	1.11 ± 0.11 ^a^	1.00 ± 0.00 ^a^
	CP	1.29 ± 0.29 ^a^	1.29 ± 0.29 ^a^	1.50 ± 0.20 ^a^	1.65 ± 0.28 ^a^
	CS	1.00 ± 0.00 ^a^	1.00 ± 0.00 ^0a^	1.23 ± 0.17 ^a^	3.06 ± 1.33 ^b^
sucking predators	CC	2.56 ± 0.45 ^a^	1.49 ± 0.11 ^a^	2.56 ± 0.45 ^a^	1.49 ± 0.11 ^a^
	CP	3.09 ± 0.40 ^a^	1.71 ± 0.12 ^a^	3.09 ± 0.40 ^a^	1.71 ± 0.12 ^a^
	CS	2.98 ± 0.52 ^a^	1.47 ± 0.11 ^a^	2.98 ± 0.52 ^a^	1.47 ± 0.11 ^a^
all herbivores	CC	2.14 ± 0.17 ^a^	3.68 ± 0.51 ^ab^	2.65 ± 0.31 ^a^	2.31 ± 0.33 ^a^
	CP	2.77 ± 0.28 ^b^	3.98 ± 0.53 ^a^	2.87 ± 0.31 ^b^	2.38 ± 0.21 ^a^
	CS	2.38 ± 0.22 ^a^	3.23 ± 0.42 ^b^	2.26 ± 0.20 ^a^	2.20 ± 0.22 ^a^
chewing herbivores	CC	1.52 ± 0.12 ^ab^	1.52 ± 0.12 ^ab^	1.71 ± 0.20 ^a^	1.68 ± 0.18 ^a^
	CP	1.88 ± 0.20 ^a^	1.88 ± 0.20 ^a^	1.66 ± 0.15 ^a^	1.74 ± 0.17 ^a^
	CS	1.49 ± 0.20 ^b^	1.49 ± 0.20 ^b^	1.41 ± 0.1 ^a^	2.07 ± 0.25 ^a^
sucking herbivores	CC	2.37 ± 0.22 ^a^	4.26 ± 0.62 ^ab^	3.22 ± 0.47 ^a^	2.67 ± 0.51 ^a^
	CP	3.13 ± 0.38 ^b^	4.35 ± 0.60 ^a^	3.48 ± 0.45 ^b^	2.91 ± 0.35 ^a^
	CS	2.68 ± 0.28 ^ab^	3.55 ± 0.48 ^b^	2.73 ± 0.31 ^a^	2.28 ± 0.31 ^a^
parasitoids	CC	1.32 ± 0.12 ^a^	1.11 ± 0.06 ^a^	1.23 ± 0.11 ^a^	1.09 ± 0.09 ^a^
	CP	1.24 ± 0.07 ^a^	1.27 ± 0.14 ^a^	1.24 ± 0.08 ^a^	1.12 ± 0.07 ^a^
	CS	1.20 ± 0.06 ^a^	1.09 ± 0.05 ^a^	1.19 ± 0.08 ^a^	1.06 ± 0.06 ^a^
spiders	CC	1.10 ± 0.05 ^a^	1.17 ± 0.07 ^a^	1.40 ± 0.13 ^a^	1.19 ± 0.14 ^a^
	CP	1.14 ± 0.07 ^a^	1.04 ± 0.04 ^a^	1.24 ± 0.07 ^a^	1.12 ± 0.08 ^a^
	CS	1.12 ± 0.07 ^a^	1.53 ± 0.21 ^a^	1.25 ± 0.07 ^a^	1.00 ± 0.00 ^a^

Mean abundance of arthropods (Mean ± SE), was calculated by averaging the number of the arthropod individuals found per each block, date, and treatment. Means ± SE that share the same letter(s) are not different among treatments at α = 0.05, based on multiple means comparisons (by computing estimated marginal means).

**Table 3:** Mean abundances of all arthropods and their main feeding guilds captured in the pitfall trap samples in soybean in 2018 at two field sites, Beltsville [Central Maryland (CM)] and Keedysville [Western Maryland (WM)], across three post-harvest treatments following corn harvest. For the three treatments, CC denotes cover crop planted in corn residue/stubble; CP represents chisel plowed (conventional tillage) plots; and CS (corn stubble) no action. Mean values and standard errors (Mean ± SE) and results of statistical comparisons via fitted GLMMs are reported.

Feeding Guild	Treatment	CM	WM
		Mean ± SE	Mean ± SE
all arthropods	CC	5.04 ± 1.00 ^a^	4.52 ± 0.39 ^a^
	CP	5.33 ± 1.33 ^b^	3.92 ± 0.78 ^b^
	CS	3.30 ± 0.50 ^c^	3.96 ± 0.36 ^c^
all predators	CC	3.61 ± 0.78 ^a^	4.47 ± 1.14 ^a^
	CP	5.80 ± 2.66 ^a^	2.35 ± 0.47 ^b^
	CS	4.17 ± 1.00 ^b^	2.24 ± 0.29 ^b^
chewing predators	CC	4.13 ± 1.01 ^a^	3.12 ± 0.48 ^a^
	CP	6.00 ± 2.80 ^a^	2.11 ± 0.40 ^b^
	CS	4.56 ± 1.09 ^b^	2.26 ± 0.33 ^b^
sucking predators	CC	2.00 ± 0.52 ^a^	13.40 ± 7.49 ^a^
	CP	2.00 ± NA *^,b^	4.50 ± 3.50 ^a^
	CS	1.00 ± 0.00 ^c^	2.17 ± 0.54 ^b^
all herbivores	CC	2.38 ± 0.35 ^a^	5.00 ± 1.45 ^a^
	CP	1.96 ± 0.35 ^b^	12.73 ± 8.47 ^b^
	CS	1.85 ± 0.29 ^b^	5.43 ± 1.69 ^a^
chewing herbivores	CC	2.73 ± 0.44 ^a^	5.79 ± 1.71 ^a^
	CP	2.32 ± 0.50 ^b^	20.22 ± 13.84 ^b^
	CS	1.67 ± 0.39 ^b^	8.44 ± 2.74 ^c^
sucking herbivores	CC	1.30 ± 0.21 ^a^	1.20 ± 0.20 ^a^
	CP	1.22 ± 0.15 ^a^	1.50 ± 0.34 ^a^
	CS	2.13 ± 0.43 ^b^	1.42 ± 0.23 ^a^
parasitoids	CC	1.17 ± 0.17 ^a^	1.00 ± 0.00 ^a^
	CP	1.11 ± 0.11 ^a^	1.09 ± 0.09 ^a^
	CS	1.11 ± 0.11 ^a^	1.13 ± 0.13 ^a^
spiders	CC	2.51 ± 0.55 ^a^	1.65 ± 0.20 ^a^
	CP	4.53 ± 1.42 ^b^	2.00 ± 0.36 ^a^
	CS	2.69 ± 0.54 ^a^	2.13 ± 0.39 ^a^

Mean abundance of arthropods (Mean ± SE), was calculated by averaging the number of the arthropod individuals found per each block, date, and treatment. Means ± SE that share the same letter(s) are not different among treatments at α = 0.05, based on multiple means comparisons (by computing estimated marginal means). * This SE output is tentatively due to the small sample size.

**Table 4:** Percentage of stink bug egg mortality due to parasitism and predation, and proportion of hatched individuals in soybean during 2017 and 2018, at one field site, Beltsville [Central Maryland (CM)], across three post-harvest treatments following corn harvest. For the three treatments, CC denotes cover crop planted in corn residue/stubble; CP represents chisel plowed (conventional tillage) plot; and CS (corn stubble) no action.

		2017	2018
Egg Fate	Treatment	Mean ± SE *	Mean ± SE
parasitism	CC	19.69 ± 7.00 ^a^	17.47 ± 6.06 ^a^
	CP	19.83 ± 5.82 ^a^	33.83 ± 9.59 ^b^
	CS	15.06 ± 5.54 ^b^	49.67 ± 25.52 ^c^
predation	CC	15.19 ± 5.79 ^a^	25.00 ± 8.44 ^a^
	CP	12.50 ± 5.10 ^a^	4.17 ± 1.58 ^b^
	CS	16.06 ± 6.48 ^a^	2.80 ± 2.04 ^c^
hatch	CC	75.25 ± 23.18 ^a^	92.80 ± 32.70 ^a^
	CP	58.67 ± 17.87 ^b^	89.22 ± 27.58 ^b^
	CS	61.53 ± 16.26 ^b^	94.87 ± 30.85 ^a^

* Means ± SE that share the same letter(s) are not different among treatments at α = 0.05, based on multiple means comparisons (by computing estimated marginal means).

**Table 5:** Percentage of stink bug egg mortality due to parasitism and predation, and proportion of hatched individuals in soybean plots during 2017 and 2018, at one field site, Beltsville [Central Maryland (CM)]. Data are compared between species. Mean values and standard errors (Mean ± SE) and results of statistical comparisons via fitted GLMMs and LMMs are reported.

		2017		2018
Egg Fate	Insect Species **	Mean ± SE	Insect Species	Mean ± SE
parasitism	BMSB	0.00 ± 0.00 ^a^	BMSB	0.34 ± 0.14 ^abc^
	BSB	0.86 ± 0.06 ^b^	BSB	0.81 ± 0.05 ^bc^
	GSB	0.16 ± 0.03 ^a^	GSB	0.18 ± 0.03 ^a^
	HB	1.00 ± 0.00	KB	0.00 ± 0.00
	KB ***	0.00 ± 0.00	RSSB	1.00 ± 0.00
	SSB	1.00 ± 0.00 ^b^	SSB	0.67 ± 0.33 ^abc^
predation	BMSB	0.72 ± 0.19 ^a^	BMSB	0.20 ± 0.09 ^a^
	BSB	0.05 ± 0.02 ^b^	BSB	0.06 ± 0.03 ^ab^
	GSB	0.16 ± 0.03 ^b^	GSB	0.09 ± 0.02 ^b^
	HB	0.00 ± 00.0	KB	0.00 ± 0.00
	KB	0.01 ± 0.01	RSSB	0.00 ± 0.00
	SSB	0.00 ± 0.00 ^b^	SSB	0.00 ± 0.00 ^ab^
hatch	BMSB	0.25 ± 0.19 ^abc^	BMSB	0.32 ± 0.11 ^ab^
	BSB	0.03 ± 0.03 ^bc^	BSB	0.07 ± 0.03 ^ab^
	GSB	0.59 ± 0.04 ^a^	GSB	0.65 ± 0.04 ^b^
	HB	0.00 ± 0.00	KB	0.87 ± 0.03
	KB	0.77 ± 0.04	RSSB	0.00 ± 0.00
	SSB	0.00 ± 0.00 ^bc^	SSB	0.33 ± 0.33 ^ab^

** Insect species: the kudzu bug (KB), *Megacopta cribraria*; the brown marmorated stink bug (BMSB), *Halyomorpha halys*; the brown stink bug (BSB), *Euschistus servus*; the green stink bug (GSB), *Chinavia hilaris*; the spined soldier bug (SSB), *Podisus maculiventris*. *** KB was analyzed separately; the red-shouldered stink bug (RSSB), *Thyanta custator*, and Harlequin bug (HB), *Murgantia histrionica* were not included in this analysis due to their small sample size. Means ± SE that share the same letter(s) are not different among treatments at α = 0.05, based on multiple means comparisons (by computing estimated marginal means).

**Table 6:** Percentage of Kudzu bug egg mortality due to predation and unknown factors, and proportion of hatched individuals in soybean during 2017 and 2018, at one field site, Beltsville [Central Maryland (CM)], across three post-harvest treatments following corn harvest. For the three treatments, CC denotes cover crop planted in corn residue/stubble; CP represents chisel plowed (conventional tillage) plot; and CS (corn stubble) no action.

		2017	2018
Egg Fate	Treatment	Mean ± SE *	Mean ± SE
Mortality	CC	9.45 ± 7.55 ^a^	5.79 ± 2.52 ^a^
	CP	16.04 ± 4.67 ^a^	9.75 ± 2.13 ^a^
	CS	19.18 ± 8.54 ^a^	6.83 ± 1.84 ^a^
Hatch	CC	78.68 ± 11.09 ^a^	76.08 ± 7.66 ^a^
	CP	77.29 ± 4.18 ^a^	90.25 ± 2.13 ^a^
	CS	75.06 ± 7.45 ^a^	93.17 ± 1.84 ^a^

* Means ± SE that share the same letter(s) are not different among treatments at α = 0.05, based on multiple means comparisons (by computing estimated marginal means).

**Table 7:** Soybean yield in 2017 and 2018 at two field sites, Beltsville [Central Maryland (CM)] and Keedysville [Western Maryland (WM)], across three post-harvest treatments following corn harvest. For the three treatments, CC denotes cover crop planted in corn residue/stubble; CP represents plots that were chisel plowed (conventional tillage); and CS (corn stubble) no action.

Treatment	2017	2017	2018	2018
	CM	WM	CM	WM
	Mean ± SE (kg/ha)	Mean ± SE (kg/ha)	Mean ± SE (kg/ha)	Mean ± SE (kg/ha)
CC	5345.0 ± 222.9 ^a^	3161.0 ± 667.7 ^a^	4626.7 ± 167.1 ^a^	4613.8 ± 174.1 ^a^
CP	5603.2 ± 194.9 ^a^	5299.2 ± 136.5 ^b^	4277.9 ± 265.8 ^a^	4343.2 ± 54.7 ^a^
CS	5440.4 ± 524.0 ^a^	5283.8 ± 262.7 ^b^	4254.7 ± 158.3 ^a^	4260.2 ± 65.9 ^a^

Means ± SE that share the same letter(s) are not different among treatments at α = 0.05, based on multiple means comparisons (by computing estimated marginal means).

## Data Availability

The data presented in this study are available on request from the corresponding author.
